# Extended fragrance ingredients surveillance study (EFISS)—protocol for a clinical surveillance study on contact allergy to 7 fragrance materials in widespread use but hitherto not systematically patch tested

**DOI:** 10.1007/s00403-025-04286-9

**Published:** 2025-05-23

**Authors:** Wolfgang Uter, Ana Carolina Figueiredo, Anna Belloni Fortina, John Bourke, Jim Bridges, Margarida Gonçalo, Stamatis Gregoriou, Claudia Lang, Suzana Ljubojević Hadžavdić, Joseph Huggard, Marléne Isaksson, Karl-Heinz Jöckel, Ian Kimber, Dimitra Koumaki, Elena Pezzolo, Thomas Rustemeyer, Marie L. A. Schuttelaar, Cecilia Svedman, Matthias Vey, Ian White, Anna Zambello, Magnus Bruze

**Affiliations:** 1 Department of Medical Informatics, Biometry and Epidemiology Friedrich-Alexander Universität Erlangen/Nürnberg, Waldstr. 6, 91054 Erlangen, Germany; 2 Department of Dermatology, University Hospital, Coimbra Local Health Unit, Coimbra, Portugal; 3 Pediatric Dermatology Unit, Department of Medicine, University of Padova, Padova, Italy; 4 Dermatology Department, South Infirmary Victoria University Hospital, Cork, Ireland; 5 Research for Sustainability, University of Surrey, Guildford, UK; 6 Dermatology, Faculty of Medicine, University of Coimbra, Coimbra, Portugal; 7 Department of Dermatology and Venereology, Andreas Sygros Hospital, Medical School of Athens, National and Kapodistrian University of Athens, Athens, Greece; 8 Department of Dermatology, University Hospital Zürich, Zürich, Switzerland; 9 Department of Dermatology and Venereology, University Hospital Center Zagreb, University of Zagreb School of Medicine, Zagreb, Croatia; 10 The Huggard Consulting Group, S.A.R.L, Itzig, Luxembourg; 11 Department of Occupational and Environmental Dermatology, Skåne University Hospital Malmö, Malmö, Sweden; 12 Medical Faculty, University of Duisburg-Essen, University Hospital Essen, Essen, Germany; 13 Faculty of Biology, Medicine and Health, University of Manchester, Manchester, UK; 14 Department of Dermatology and Venereology, University Hospital of Heraklion, Heraklion, Crete, Greece; 15 Department of Dermatology, San Bortolo Hospital, Vicenza, Italy; 16 Department of Dermatology-Allergology, Amsterdam University Medical Centers, Amsterdam, The Netherlands; 17 Department of Dermatology, University Medical Center Groningen, Groningen, The Netherlands; 18 Department of Occupational and Environmental Dermatology, Lund University, Skåne University Hospital, Malmö, Sweden; 19 IFRA VP Scientific Affairs and IDEA Management Team, Brussels, Belgium; 20 St John’s Institute of Dermatology, Guy’s Hospital, London, UK

**Keywords:** Contact allergy, Cross-sectional study, Epidemiological surveillance, Fragrance ingredients

## Abstract

Contact allergy (CA) is not uncommon in the population, including to various fragrance allergens. If not diagnosed correctly, allergic contact dermatitis (ACD) may ensue, because targeted allergen avoidance is not possible. The primary objective of the study is to estimate the prevalence of CA to seven fragrance materials in patients with suspected ACD across Europe. Based on the outcome, a conclusion will be drawn as to whether present risk management regarding maximum recommended concentrations of each of these, based on quantitative risk assessment (QRA2), is adequate. The planned study is a surveillance study based on consecutive patients, patch tested in 10 European departments of dermatology with a series of allergens as indicated by their personal history, including the European baseline series, supplemented with the seven additional fragrance ingredients. The patch test procedure will follow the guideline of the European Society of Contact Dermatitis (ESCD) with additional standardization procedures. The envisaged sample size is 8100; recruitment will be in three data cycles with brief intervals allowing for descriptive interim analyses. Those patients reacting positively to any of the study allergens will be followed-up specifically to identify the source of sensitizing and/or eliciting exposure(s). Results will inform risk reassessment and subsequent risk management measures. Study results will be published in an open-access peer-reviewed scientific journal. Structured post-marketing surveillance of consumer risk of contact allergy by monitoring prevalences of positive patch test reactions in a dedicated European expert network is developed which can serve as a model for further chemicals. Important outcomes will be either a confirmation of effectiveness of risk management measures in place, or alternatively identifying aspects needing improvement (for certain cosmetic product categories). DRKS registration (DRKS00033263) 16.09.2024, mirrored at https://trialsearch.who.int

**Supplementary Information:**

The online version contains supplementary material available at 10.1007/s00403-025-04286-9.

## Background

Contact allergy (CA), or skin sensitization, is an acquired alteration of the immune system caused by small molecular weight chemicals (typically < 1000 D). These molecules, called “haptens”, penetrate the epidermal skin barrier and bind to epidermal proteins to create a complete antigen, that is then delivered to draining lymph nodes [1]. Only in some of those exposed this newly formed complete antigen will be recognised as foreign and cause an immune response. This adaptive immune response is mainly driven by T cells, but with other cells from the innate immune system having important roles in the process of initial sensitization and also during subsequent elicitation of cutaneous allergic reaction(s). The elicitation reaction causes the clinical signs and symptoms of allergic contact dermatitis (ACD), a potentially recurring disease with each subsequent contact with the inducing chemical. Over 5000 substances have been identified as haptens, i.e., having the potential to cause skin sensitization and allergic contact dermatitis [2]. Among these, fragrance substances are a common cause of ACD [3]. According to a population-based epidemiological study conducted in five European countries from 2008 to 2010, the prevalence of CA to the routinely tested screening agents fragrance mix (FM) I, FM II and *Myroxylon pereirae* resin (balsam of Peru) was 2.6, 1.9 and 0.7%, respectively. [4, 5] However, epidemiological surveillance of contact allergy mostly relies on clinical data, that is, diagnostic results with the so-called patch test applied to patients in whom ACD is suspected [6]. Owing to the evident morbidity-driven selection of patients with ACD who are patch tested, the prevalence is higher in these patients than at the population level [7–11].

CA and subsequent ACD upon sufficient eliciting exposure of the sensitized subject to the offending hapten is a significant health burden. It may compromise quality of life and in severe cases even the ability to work. For this reason, regulatory interventions and industry governance efforts focus on the prevention of CA. One example of the former in the field of cosmetic (or personal care) products is the European Union (EU) Cosmetics Regulation (EC 1223/2009). On the level of primary prevention, pre-marketing risk assessment aims at ensuring risk management measures are appropriate to minimize risk, e.g., by establishing concentration limits, often specific for certain cosmetic product types or user groups. To this end, Quantitative Risk Assessment (QRA) for skin sensitization has been developed, led by the fragrance industry with cooperation of stakeholders. QRA, presently in its second revision (QRA2), promotes safe use levels of sensitizing fragrance ingredients in different consumer product types (see [12] including current evaluation by the Scientific Committee on Consumer Safety [SCCS] [13]). “Safe use” is a requirement based on the EU Cosmetics Regulation preamble that cosmetic products should be safe for the consumer under reasonable and foreseeable conditions of use. Currently, QRA2-derived limits are incorporated in the Standards of the International Fragrance Association (https://www.ifrafragrance.org/, last accessed 2025-04-07). At this time, QRA, although based on sound scientific principles, remains a model that is yet to be finally assessed for efficacy.

It is therefore important to monitor, post-marketing, the success of primary prevention, as pre-marketing risk assessment may occasionally fail to determine a safe level. To this end, the epidemiological surveillance of CA incidence to a specific fragrance (or other) substance in patients patch tested for suspected ACD provides an important, and possibly the most important, contribution to the safety of ingredients used in consumer products. This includes a follow-up on increases in exposure or changes to patterns of exposure and any associated changes to specific CA morbidity. Thereby, different objectives are met: (i) industry stewardship with regard to closely monitoring the safety of ingredients introduced to the market, (ii) evidence provided to regulatory bodies and their associated expert panels regarding risk re-assessment and subsequent adaptation of risk management, and (iii) patient benefit from the expanded diagnostic scope in terms of “test allergens” under scrutiny that are applied to those who may have been exposed to them and sensitized.

Different study options to achieve such surveillance were explored during the course of three separate multi-stakeholder workshops under the project IDEA (http://www.ideaproject.info/). An initial concept was a cohort study of 12,000 immunologically naïve (non-sensitized) volunteers followed-up over a period of at least 10 years, with follow-up patch testing every 3 years, supported by a suitable questionnaire to determine exposure. However, specific CA to a fragrance substance which has been formulated to be presumably non-sensitizing in cosmetic usage, following QRA [14], would probably be very infrequent. Following this assumption, a cohort study was considered inappropriate because it would be unsuitable for (very) low incidence outcomes. Moreover, ethical concerns were raised with regard to repeatedly patch testing non-sensitized, asymptomatic individuals. Eventually, a clinical surveillance study run over an extended period, i.e., involving patients who will be routinely patch tested for suspected ACD, was considered the most effective and most workable approach. This will involve fragrance materials hitherto not tested systematically in addition to standard diagnostic care using established patch test allergens and individual patient materials (suspected consumer products including e.g. perfumes, deodorants etc.). This study concept termed “Extended Fragrance Ingredients Surveillance Study (EFISS)” is to explore the feasibility and usefulness of targeted post-marketing surveillance of the risk of CA to the selected fragrance compounds based on (i) a structured, standardised approach and (ii) a study network of experienced European dermatological clinics [15].

## Methods

### Administration and ethics

The International Fragrance Association (IFRA) is the financial sponsor of the study that is carried out under the IDEA (International Dialogue for the Evaluation of Allergens; https://ideaproject.info/) project with oversight from a Supervisory Group (https://ideaproject.info/governance). IDEA is a long-term, multi-stakeholder, expert-led program that seeks to advance consumer protection by taking a responsible approach to addressing fragrance CA. Following agreement from the sites to participate, the necessary contracts will be established with each of the sites, covering supply of materials and payments on a per-patient basis. Insurance will be obtained and support given to sites in making any necessary ethics submissions. Data sharing agreements between participating departments and the study centre will be established to address GDPR (General Data Protection Regulation) requirements. These are based on standardized technical and organizational measures and on informed patient consent to the transmission of anonymous or pseudonymous study data, respectively, depending on the local mode of study documentation. As part of the initiation process, training will be carried out with the sites on procedures, data collection, and entry into the CRF/eCRF. A hotline to the project managers will be put in place, as was the case in the EFISS pilot study [15], to allow rapid contact and follow-up on any positive results from the additional study materials. The advice of the EFISS Steering Committee will also be sought as to potential actions to be taken. The findings of the three cycles of the study will be submitted for publication in international peer-reviewed journals.

The study has been registered with the German Clinical Trials Register (DRKS), registration number DRKS00033263. DRKS is a WHO-accepted primary study register and the registration is mirrored onto https://trialsearch.who.int. Ethical approval was sought from every institutional review board associated with the clinics involved.

### Patch testing

Patch testing is the standard procedure to diagnose contact allergy (CA) [16], including to fragrance substances. The arrays of haptens applied for patch testing are organised in so-called test series of varying length, which reflect typical exposures (e.g. “hair cosmetics series”, “plastics/glues series”, etc.). A so-called “baseline series” comprises haptens found to be important to the majority of ACD patients; this is normally tested in all consecutively patch tested patients. This baseline series internationally includes fragrance mix (FM) I, FM II, and *Myroxylon pereirae* resin (balsam of Peru, BOP), c.f the European baseline series [17]. The procedure of patch testing has achieved a high level of standardization; there are guidelines by the European Society of Contact Dermatitis (ESCD) [16] followed in the EFISS. A refinement of this quality standard is that by using a clear definition of the threshold between a doubtful and a weak allergic reaction and by employing a comprehensive protocol, quality coordination, and on-site monitoring, there is further potential to reduce intra- and inter-clinic variations. The protocol also requires regular testing of the performance of the test personnel filling the chambers with test weighing of chamber doses.

The test materials include five synthetic fragrance ingredients, see Table 1. These five were selected in dedicated IDEA activities by non-industry specialists. As selection criteria, they had either to be included in the list of “established contact allergens in humans” compiled in the SCCS Fragrance Opinion (SCCS/1459/11) [3], or experimental research and assessment by the Research Institute for Fragrance Materials (RIFM) was indicating sensitization potential [18–23]. Moreover, their volume of use in various product types should be moderate to high (approximately 5000–280000 kg/year based on 2015 data), leading to relevant consumer exposure. Detailed volume of use data are not published, but banded information is accessible via the Elsevier Fragrance Materials Safety Assessment centre (https://fragrancematerialsafetyresource.elsevier.com/ last accessed 2025-04-07), where safety assessments of individual materials can be found either by searching for CAS numbers or by name. In effect, the selection criteria—clear evidence of sensitization potential combined with considerable exposure—should ensure that a detectable signal will result from epidemiological surveillance, in case risk management has hitherto not been sufficiently conservative.

**Table 1:** The study fragrance materials

Substance/extract	CAS No	EC3-value	NESIL (μg/cm^2^)	Test dose (mg/cm^2^)	Supplier
Furaneol [4-hydroxy-2,5-dimethyl-3(2H)-furanone]	3658-77-3	Mean: 450 μg/cm^2^	590	0.49	Firmenich, Geneva, Switzerland
*Trans*−2-Hexenal	6728-26-3	Mean: 1012 μg/cm^2^	18	0.02	Givaudan, Vernier, Switzerland
4,8-Dimethyl-4,9-decadienal	71077-31-1	n.a	550	0.46	IFF
Longifolene	475-20-7	1.75% to 31.4%	3500	2.81	IFF
Benzaldehyde	100-52-7	> 6250 μg/cm^2^	590	0.49	Firmenich, Geneva, Switzerland
*Evernia prunastri* (oakmoss abs.) “low”	90028-68-5, 68917-10-2, 9000-50-4	–	700^a^	0.80	Robertet, Grasse, France
*Evernia furfuracea* (treemoss abs.) “low”	90028-67-4, 68648-41-9, 68917-40-8	–	700^a^	0.80	Robertet, Grasse, France

“Low” denotes trace level content (< 100 ppm in extract) of atranol and chloroatranol. All patch test preparations in petrolatum, EC3, eliciting concentration 3: concentration which leads to a three-fold increase (interpolated) of the stimulation index in the Local Lymph Node Assay (LLNA)

*IFF* International Flavors & Fragrances Inc., New York, USA, *n.a.* not available (no animal data), *NESIL* No Expected Sensitization Induction Level

^a^for these extracts, technical reports referenced in the respective IFRA standards of the 43rd amendment (see https://ifrafragrance.org/safe-use/standards-documentation, last accessed 2024-11-12) are available

All these five audit substances are synthetic fragrance materials but can also be present in natural extracts. They are widely used in consumer products for almost 40 years. [30] Four of them, except 4,8-dimethyl-4,9-decadienal, are also used as flavours in the food industry. In the fragrance ingredient safety assessment of the RIFM, a No Expected Sensitization Induction Level (NESIL) was calculated to determine the skin sensitization safety level, see Table 1. All test materials are donated from the manufacturers shown in the table and are mixed in petrolatum (pet.; density 0.9 g/cm^2^) for patch testing purposes, standardization provided by high-performance liquid chromatography (HPLC) analysis. Moreover, stability and homogeneity of the patch test materials during the intended shelf live is verified by Chemotechnique (Vellinge, Sweden). Among the selected fragrances, only benzaldehyde 5% pet. has been previously tested in two studies from Austria and the Information Network of Departments of Dermatology (https://ivdk.org/en/), respectively, yielding 3/747 and 6/2820 positive (and 19 doubtful or irritant) reactions, respectively [24, 25].

Furthermore, *Evernia prunastri* (oakmoss) and *Evernia furfuracea* (treemoss) extracts which had been processed to reduce the content of their main allergens atranol and chloroatranol to trace levels are included in the EFISS. This is because the former quality of the mosses, used hitherto for patch testing, is disappearing from the market and being replaced by the qualities where the concentrations of atranol and chloroatranol in oakmoss as well as treemoss have been reduced to a maximum of 100 ppm, i.e., not exceeding 0.5 ppm (trace level) in the typical application in a fine fragrance. According to a study employing serial dilution testing both with conventional and low (chlor)atranol content, the latter yielded significantly lower patch test elicitation, owing to the much reduced content of these two extreme allergens [26]. Such trace levels are regarded as being compliant with the ban of atranol and chloroatranol on the EU market in August 2021 [27]. It is considered important to test the old and new qualities in parallel to collect evidence allowing to understand potential differences in reactions of sensitized patients. In addition to above study materials, the full scope of 26 fragrance allergens presently requiring ingredient labelling in the EU are tested in the consecutive patients, along with the screening mixes. Besides ensuring in-depth diagnostic coverage, the analysis of patch test results with these additional fragrance allergens, including cross-reactivity, will be a secondary objective of the EFISS.

In a pilot study to the EFISS [15], the five ingredients mentioned first were tested in patients with suspected allergic contact dermatitis to determine a suitable patch test concentration for each (see Table 1), following a standardized protocol along with the oakmoss and tree moss with low atranol and chloroatranol content at established test concentrations [28]. The pilot study also tested the functioning of the initial protocol as a basis for developing the final main EFISS study protocol.

### Late-appearing patch test reactions

Late-appearing patch test reactions, defined as positive test reactions appearing first on day (D) 10 or later after the application of the tests, may occur. These reactions represent either induction of sensitization due to the patch test procedure (active sensitization) or a positive reaction appearing late in a pre-sensitized individual. Evidence of active sensitization would need a timely reconsideration (lowering) of the patch test concentration used. Hence the distinction between the two above aetiologies is important for the safety of the study procedure.

**Fig. 1:**
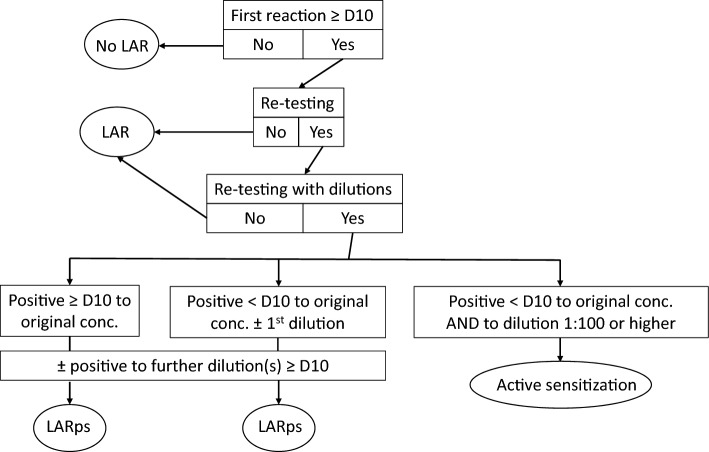
Flow-chart for classification of a late-appearing reaction (LAR). *conc*. patch test concentration, *D* days after the start of patch test exposure, *LARps* LAR in a pre-sensitized individual, ±  criterion may or may not be met

Such reactions are rare and did not appear during the pilot study, including with the very concentrations used in the main study [15]. As regards fragrance materials, late-appearing reactions have been reported representing both active sensitization [29] and late reactions in pre-sensitized individuals. Although a late-appearing reaction may thus represent active sensitization, it must never automatically be interpreted as proof of active sensitization, even if a re-test with the same test preparation will result in a positive reaction within one week during the re-test. Generally, the test reactivity in terms of the lowest dose/concentration eliciting a positive test reaction may vary considerably [30–35]. Late-appearing reactions to many sensitizers have been reported as not being due to active sensitization [36–42]. If a late-appearing reaction is noted in the present surveillance study, re-testing should be performed to confirm/rule out active sensitization, the most serious adverse reaction in patch testing. The re-test should be performed with the same test preparation, same dose in mg/cm^2^, and same test methodology, as well as testing with dilutions of the sensitizer. Besides reading of the re-test on D 3/4 and D 7, further readings, possibly by photos taken by the study participant, should be performed on D 10 and after 2 and 3 weeks (D 14 and D 21). In this study, the following definitions will be used for classification of the initial (index) patch test reaction (Fig. 1):

- Late-appearing reaction—(i) when there is no re-test or (ii) when the re-test is only performed with the petrolatum preparation (same concentration) of the suspected material. This is independent of how many days any reaction appears during re-testing.
- Late-appearing reaction in a pre-sensitized individual—when the re-test includes a dilution series and (i) the reaction to the petrolatum preparation again appears late or (ii) appears within one week at the re-test, possibly together with a reaction to the first dilution, but without any reactions to the other dilutions during the first week after the application of the re-test. Positive reactions to further dilutions appearing on D 10 or later are compatible with this definition.
- Active sensitization—when, at re-testing, there are positive reactions within one week to the original petrolatum preparation of the material and to the material diluted 100 times or more [43].

If more than one case of active sensitization occurs, patch testing with the responsible fragrance material will be stopped until a careful evaluation of the evidence has been performed. It may be decided that the fragrance material will thereafter be tested at a lower dose/area (concentration) [28].

### Clinical sampling

Eligibility covers all patients in whom a patch test is planned for the diagnosis of suspected CA/ACD, age 18 years and above, in terms of the “consecutive patients” referred to above. No further study-related exclusion criteria will be employed beyond those generally recommended in the ESCD patch test guideline [16]. Patients sign institution-specific informed consent (i) to be patch tested also with the additional study materials, in addition to routine information on the procedure used in the institution and (ii) to have their data recorded, pooled, and analysed in a pseudonymous or anonymous fashion, according to departmental standards and compliant to GDPR requirements. It is stressed that publication of results will show no identifiable profiles of study participants, e.g. by increasing granularity of individual information, such as age by employing 5 year age groups. The participating patch test clinics are shown in Table 2. In case approval of contribution to the present study is not provided by an eligible patient, this will be documented separately in an anonymous fashion to be able to derive information on the study participation rate. The study is planned for three cycles over a duration of approximately 5 years of the EFISS. Interim analyses at the end of each cycle will address possible emergence of an important contact allergy problem, or any observations with patch testing the study materials (Table 1). Over these three cycles, the study will involve approximately 8100 consecutive dermatitis patients that will be patch tested in 10 centres in eight European countries. This sample size will allow for a relatively precise prevalence estimation in case of no event (0, 95 CI 0–0.046%) [44] or a low-level prevalence event (e.g., 0.51, 95 CI 0.36–0.69%), and is considered, at the same time, feasible to achieve.

**Table 2:** Participating departments

Institution	Investigators
Dermatologische Klinik, Universitätsspital Zürich, Switzerland	Claudia Lang
University of Athens, Andreas Sygros Hospital, Greece	Stamatis Gregoriou
University Hospital of Heraklion, Crete, Greece	Dimitra Koumaki
Department of Dermatology and Venereology, University Hospital Center Zagreb, University of Zagreb School of Medicine, Croatia	Suzana Ljubojević Hadžavdić
South Infirmary Victoria University Hospital Cork, Ireland	John Bourke
Department of Medicine DIMED, University of Padova, Padova, Italy	Anna Belloni Fortina, Anna Zambello
Department of Dermatology, AULSS8 Berica, Ospedale San Bortolo, Vicenza, Italy	Elena Pezzolo
University Medical Center Groningen, Department of Dermatology, Groningen, The Netherlands	Marie-Louise A. Schuttelaar
Serviço de Dermatologia Centro Hospitalar e Universitário de Coimbra, Portugal	Margarida Gonçalo, Ana Carolina Figueiredo
Skane University Hospital and Department of Occupational and Environmental Dermatology, Lund University Malmö, Sweden	Cecilia Svedman, Magnus Bruze

### Data capture and analysis

Primary data capturing will be performed with a paper case record form (CRF; see Appendix A), except in those departments who have fully implemented the study documentation in their regular patch test or other general clinical documentation software in terms of an electronic CRF (eCRF). Patients’ history, their relevant demographic and clinical characteristics, and patch test results are thus documented for every study participant. In case of a positive (allergic) reaction to any of the seven test substances, an additional form (see Appendix B) on detailed information (patch test reaction pattern, product(s) suspected to have caused ACD) is used and transmitted to the study management unit established under IDEA. In addition, a hotline to the study management is available, which the clinics are encouraged to use to facilitate a speedy response. This response includes but is not limited to determining from manufacturers of the suspected product(s) whether the diagnosed allergen is an ingredient of the product(s). Concerning four out of the five chemically defined materials, this is necessary to thus ascertain the presence (or absence) of the substances in the cosmetic products regarded as potential causes of ACD, as these materials have not yet been covered by cosmetic product labelling requirements (EC 1223/2009). By contrast, labelling on cosmetic products has already been established in Europe for benzaldehyde, which will be included in the list of fragrance ingredients to be put on the label in the course of the study period [45]. Labelling has also been in place for about two decades, for a number of other fragrance ingredients, e.g. those assembled in the two screening mixes used in the patch test baseline series, and including *E. prunastri* and *E. furfuracea*. The collective information on the presence of the hapten(s) found positive in the patch test is considered during the evaluation of the so-called clinical relevance, i.e., whether the CA diagnosed corresponds to ACD upon previous contact with products containing the substance.

Further processing of data is electronic, with local data entry and central pooling of results. The final outcome of the dedicated assessment (presence in suspected product, eventual judgment on clinical relevance) is also transmitted from the data management unit to the data centre at Erlangen University from those departments where it is not already covered by use of the dedicated online documentation system.

Post cycle interim as well as final analyses address the overall frequency of positive (allergic) reactions to each of the audit substances as primary endpoint. Secondary endpoints include (i) gender- and age-stratified results; (ii) geographically/department-wise stratified results; (iii) time trend analyses, as far as feasible; (iv) co-reactivity between audit substances and other fragrance ingredients, respectively, (v) detailed reporting of the outcomes of clinical relevance assessment of the audit substances, (vi) presentation of the patch test reaction pattern, i.e., occurrence of irritant or doubtful reactions, and the distribution of different grades of positive reactions (+ , +  + , or +  + +). Moreover, quality control results are analysed and presented. Additional secondary analyses may be added in the course of the study, e.g. using the participants testing positive to those of the study substances also used as flavours vs. those negative for a nested case–control study addressing the possible role of food flavouring in the elicitation of systemic allergic dermatitis. The R statistical software (https://www.r-project.org/) will be used for data management and analysis. Generally, the reporting will follow the standards of the STROBE checklist for cross-sectional studies [46].

## Discussion

The prevalence of CA to the seven fragrance test substances, including two natural extracts with reduced content of the main allergens, respectively, is yet unknown, even though exposure at least to the five synthetic fragrances is extensive, and a clear sensitization potential has been found. It is believed that information on the frequency of contact allergy in a broadly based European clinical sample, will provide important new evidence to the field of clinical CA epidemiology. Beyond this primary objective, the estimated prevalences will also serve as a benchmark for the success or otherwise of QRA-driven risk management, i.e. maximum safe use concentrations determined for different fragrance ingredients in different consumer product types which are in scope of the IFRA standards. Ideally, no cases of CA will be observed, which would allow to suggest that the risk management worked as expected for the tested substances, with the uncertainty indicated by the upper 95% confidence interval to a proportion of 0%, given the final sample size tested.

However, some cases of CA are expected, in view of the potential of the EFISS main study, by virtue of its sample size, to include even highly susceptible and/or highly exposed consumers who became sensitized and patch test positively. According to the preliminary results of the pilot study, when testing 149 patients with the same concentrations of the audit materials as will be used in this main study, no positive reactions were observed, giving an upper 95% confidence interval of 1.99% [15]. This finding could be interpreted as indicating that “up to 2%” allergic reactions may be expected—acknowledging that the spectrum of participating clinics has been expanded since, which may introduce additional variation.

Generally, a very low level of contact allergy diagnosed when patch testing consecutive patients could be regarded as “acceptable”, following the rationale of Thyssen et al. presented in 2009, based on the CE-DUR model [47, 48]: Extrapolated from clinical patch test data on such patients, the authors suggest that between 0.02 and 0.1% positive patch tests to a substance would correspond to a “low level epidemic” with at least 1 in 10,000 persons in the general population being sensitized. Consequently, between 0.002 and 0.01% would correspond to at least 1 in 100,000 (but less than 1 in 10,000) persons in the general population being sensitized. Such benchmarkingevidently needs sufficient discussion and general acceptability to be used in (self-) regulatory practice.

The patients identified in this study to be sensitized will have profited from the specifically designed counselling included in this study, that is, by receiving advice regarding their specific contact allergen(s) and also on products containing these or not. This is very relevant in a situation where full declaration of all fragrance ingredients has not been achieved yet (except for benzaldehyde, referring to the scope of allergens included in the study) [3]. From the perspective of effectiveness of QRA-driven risk management, anonymous information on exposure patterns of patients found sensitized can help to refine QRA and risk management based on QRA.

A third, public health objective of the EFISS is to examine the feasibility of, and usefulness of outcomes from, such post-marketing CA surveillance focused on a set of substances of interest. Hence, the study results, including possible challenges encountered, can serve as a model for similar future studies, also beyond the context of cosmetic products, e.g. targeting sensitizing ingredients of work materials. The aims as already introduced above would be the same: (i) improved industry stewardship, (ii) the availability of timely evidence on the significance of a CA issue with ingredients in consumer or occupationally use products, including for regulatory purposes, and (iii) enhanced patient benefit by providing up-to-date diagnostic coverage of contact allergens.

## Supplementary Information

Below is the link to the electronic supplementary material.

Supplementary file1 (PDF 967 KB)

Supplementary file2 (PDF 416 KB)

## Data Availability

The present publication itself is not associated with actual data, being a study protocol. Regarding the later study, public availability of clinical (anonymized) data is not envisaged, as for this no consent is sought from included patients.

## Abbreviations

ACD: Allergic contact dermatitis
CA: Contact allergy
CE-DUR: Clinical epidemiology-drug utilisation research
(e)CRF: (Electronic) case record form
D: Day following start of patch test exposure
ESCD: European society of contact dermatitis
FM: Fragrance mix (as patch test preparation)
IDEA: International dialogue for the evaluation of allergens (https://ideaproject.info/)
IFRA: International fragrance association
GDPR: General data protection regulation
HPLC: High-performance liquid chromatography
pet.: Petrolatum (Vaseline) as patch test preparation vehicle
QRA (2): Quantitative risk assessment (version 2)
RIFM: Research Institute for Fragrance Materials
SCCS: Scientific committee on consumer safety (of the EU commission)

## References

[1] Johansen JD, Bonefeld CM, Schwensen JFB, Thyssen JP, Uter W (2022) Novel insights into contact dermatitis. J Allergy Clin Immunol 149(4):1162–1171. 10.1016/j.jaci.2022.02.002 35183605 10.1016/j.jaci.2022.02.002

[2] de Groot AC (2022) Patch Testing—Test Concentrations and Vehicles for 5200 Chemicals, 5th edn. Acdegroot Publishing, Netherlands

[3] SCCS (Scientific Committee on Consumer Safety) (2012) Opinion on fragrance allergens in cosmetic products, 26–27 June 2012 (SCCS/1459/11).

[4] Diepgen TL, Ofenloch R, Bruze M et al (2015) Prevalence of fragrance contact allergy in the general population of five European countries: a cross-sectional study. Br J Dermatol 173(6):1411–1419. 10.1111/bjd.14151 26332456 10.1111/bjd.14151

[5] Bruze M, Mowitz M, Ofenloch R et al (2019) The significance of batch and patch test method in establishing contact allergy to fragrance mix I-EDEN fragrance study group. Contact Dermatitis 81(2):104–109. 10.1111/cod.13253 30810228 10.1111/cod.13253

[6] Uter W, Schnuch A, Giménez-Arnau A, Orton D, Statham B (2020) Databases and networks: the benefit for research and quality assurance in patch testing. In: Johansen JD, Mahler V, Lepoittevin JP, Frosch PJ (eds) Contact dermatitis, 6th edn. Springer, pp 1–16

[7] Sukakul T, Charoenpipatsin N, Svedman C, Boonchai W (2021) Prevalence, concomitant reactions, and factors associated with fragrance allergy in Thailand. Contact Dermatitis 84(3):175–182. 10.1111/cod.13723 33075139 10.1111/cod.13723

[8] Sukakul T, Bruze M, Mowitz M et al (2022) Simultaneous patch testing with fragrance markers in the baseline series and the ingredients of fragrance mixes: an update from southern Sweden. Contact Dermatitis 86(6):514–523. 10.1111/cod.14072 35152428 10.1111/cod.14072 PMC9314710

[9] Uter W, Wilkinson SM, Aerts O et al (2022) Patch test results with the European baseline series, 2019/20-Joint European results of the ESSCA and the EBS working groups of the ESCD, and the GEIDAC. Contact Dermatitis 87(4):343–355. 10.1111/cod.14170 35678309 10.1111/cod.14170

[10] Uter W, Bauer A, Belloni Fortina A et al (2021) Patch test results with the European baseline series and additions thereof in the ESSCA network, 2015–2018. Contact Dermatitis 84(2):109–120. 10.1111/cod.13704 32945543 10.1111/cod.13704

[11] Dear K, Bala H, Palmer A, Nixon RL (2021) How good is the Australian baseline series at detecting allergic contact dermatitis? Australas J Dermatol 62(1):51–56. 10.1111/ajd.13456 32914863 10.1111/ajd.13456

[12] SCCS (Scientific Committee on Consumer Safety) (2018) Opinion on Skin Sensitisation Quantitative Risk Assessment for Fragrance Ingredients (QRA2), Submission I (SCCS/1589/17).

[13] SCCS (Scientific Committee on Consumer Safety) (2024) SCCS (Scientific Committee on Consumer Safety), Opinion on Citral (CAS No. 5392–40–5, EC No. 226–394–6)-Sensitisation Endpoint, Preliminary Version of 27 March 2024, Final Version of 29 July 2024, SCCS/1666/24.

[14] Api AM, Vey M (2008) Implementation of the dermal sensitization quantitative risk assessment (QRA) for fragrance ingredients. Regul Toxicol Pharmacol RTP 52(1):53–61. 10.1016/j.yrtph.2008.05.011 18635300 10.1016/j.yrtph.2008.05.011

[15] Sukakul T, Uter W, Gonçalo M et al (2024) Results of patch testing with five fragrance materials hitherto not tested: a dose-finding study in the clinical population. Contact Dermatitis 90(6):566–573 38387040 10.1111/cod.14525

[16] Johansen JD, Aalto-Korte K, Agner T et al (2015) European society of contact dermatitis guideline for diagnostic patch testing - recommendations on best practice. Contact Dermatitis 73(4):195–221. 10.1111/cod.12432 26179009 10.1111/cod.12432

[17] Wilkinson SM, Gonçalo M, Aerts O et al (2023) The European baseline series and recommended additions: 2023. Contact Dermatitis 88(2):87–92. 10.1111/cod.14255 36443008 10.1111/cod.14255

[18] Api AM, Belsito D, Biserta S et al (2020) RIFM fragrance ingredient safety assessment, 4-hydroxy-2,5-dimethyl-3(2H)-furanone, CAS registry number 3658–77-3. Food Chem Toxicol Int J Publ Br Ind Biol Res Assoc 144(Suppl 1):111620. 10.1016/j.fct.2020.111620 10.1016/j.fct.2020.111620 32777341

[19] Api AM, Belsito D, Biserta S et al (2019) RIFM fragrance ingredient safety assessment, 4,8-dimethyl-4,9-decadienal, CAS registry number 71077–31-1. Food Chem Toxicol Int J Publ Br Ind Biol Res Assoc 130(Suppl 1):110648. 10.1016/j.fct.2019.110648 10.1016/j.fct.2019.110648 31255670

[20] Api AM, Belsito D, Biserta S et al (2020) Corrigendum to “RIFM fragrance ingredient safety assessment, 4,8-dimethyl-4,9-decadiena”, CAS registry number 71077-31-1 [Food Chem. Toxicol. 130, Suppl. 1 (2019) 110648]. Food Chem Toxicol Int J Publ Br Ind Biol Res Assoc. 141(Suppl 1):111407. 10.1016/j.fct.2020.111407 10.1016/j.fct.2020.111407 32402572

[21] Api AM, Belsito D, Biserta S et al (2019) RIFM fragrance ingredient safety assessment, longifolene, CAS Registry Number 475–20-7. Food Chem Toxicol Int J Publ Br Ind Biol Res Assoc 134(Suppl 2):110823. 10.1016/j.fct.2019.110823 10.1016/j.fct.2019.110823 31542430

[22] Api AM, Belsito D, Biserta S et al (2019) RIFM fragrance ingredient safety assessment, benzaldehyde, CAS Registry Number 100–52-7. Food Chem Toxicol Int J Publ Br Ind Biol Res Assoc 134(Suppl 2):110878. 10.1016/j.fct.2019.110878 10.1016/j.fct.2019.110878 31622729

[23] Api AM, Belsito D, Botelho D et al (2021) RIFM fragrance ingredient safety assessment, hexen-2-al, CAS Registry Number 6728–26-3. Food Chem Toxicol Int J Publ Br Ind Biol Res Assoc 156(Suppl 1):112425. 10.1016/j.fct.2021.112425 10.1016/j.fct.2021.112425 34289391

[24] Wöhrl S, Hemmer W, Focke M, Götz M, Jarisch R (2001) The significance of fragrance mix, balsam of Peru, colophony and propolis as screening tools in the detection of fragrance allergy. Br J Dermatol 145(2):268–273. 10.1046/j.1365-2133.2001.04345.x 11531790 10.1046/j.1365-2133.2001.04345.x

[25] Uter W, Geier J, Frosch P, Schnuch A (2010) Contact allergy to fragrances: current patch test results (2005–2008) from the information network of departments of dermatology. Contact Dermatitis 63(5):254–261. 10.1111/j.1600-0536.2010.01759.x 20731693 10.1111/j.1600-0536.2010.01759.x

[26] Mowitz M, Zimerson E, Svedman C, Bruze M (2013) Patch testing with serial dilutions and thin-layer chromatograms of oak moss absolutes containing high and low levels of atranol and chloroatranol. Contact Dermatitis 69(6):342–349. 10.1111/cod.12126 24102141 10.1111/cod.12126

[27] EU (2017) Commission Regulation (EU) 2017/1410 of 2 August 2017 Amending Annexes II and III to Regulation (EC) No 1223/2009 of the European Parliament and of the Council on Cosmetic Products.

[28] Bruze M, Svedman C, Andersen KE et al (2012) Patch test concentrations (doses in mg/cm2) for the 12 non-mix fragrance substances regulated by European legislation. Contact Dermatitis 66(3):131–136. 10.1111/j.1600-0536.2011.02037.x 22320667 10.1111/j.1600-0536.2011.02037.x

[29] Heisterberg MV, Vigan M, Johansen JD (2010) Active sensitization and contact allergy to methyl 2-octynoate. Contact Dermatitis 62(2):97–101. 10.1111/j.1600-0536.2009.01664.x 20136892 10.1111/j.1600-0536.2009.01664.x

[30] Björk AK, Bruze M, Engfeldt M, Nielsen C, Svedman C (2017) The reactivity of the back revisited. Are there differences in reactivity in different parts of the back? Contact Dermatitis 76(1):19–26. 10.1111/cod.12657 27593358 10.1111/cod.12657

[31] Hindsén M, Bruze M, Christensen OB (1999) Individual variation in nickel patch test reactivity. Am J Contact Dermat Off J Am Contact Dermat Soc 10(2):62–67. 10.1016/s1046-199x(99)90001-5 10.1016/s1046-199x(99)90001-5 10357713

[32] Masjedi K, Bruze M, Hindsén M, Minang J, Ahlborg N (2009) Is the variability of nickel patch test reactivity over time associated with fluctuations in the systemic T-cell reactivity to nickel? Br J Dermatol 161(1):102–109. 10.1111/j.1365-2133.2009.09182.x 19438434 10.1111/j.1365-2133.2009.09182.x

[33] Siemund I, Mowitz M, Zimerson E, Bruze M, Hindsén M (2017) Variation in aluminium patch test reactivity over time. Contact Dermatitis 77(5):288–296. 10.1111/cod.12836 28695639 10.1111/cod.12836

[34] Rosholm Comstedt L, Engfeldt M, Svedman C, Åkesson A, Hindsén M, Bruze M (2018) Variation and covariation in patch test reactivity to palladium and nickel salts. Eur J Dermatol EJD 28(5):668–676. 10.1684/ejd.2018.3423 30530435 10.1684/ejd.2018.3423

[35] Ofenloch RF, Andersen KE, Foti C et al (2023) Allergic reactivity for different dilutions of eugenol in repeated open application test and patch testing. Contact Dermatitis 89(2):95–102. 10.1111/cod.14333 37218587 10.1111/cod.14333

[36] Isaksson M, Bruze M (2003) Late patch-test reactions to budesonide need not be a sign of sensitization induced by the test procedure. Am J Contact Dermat Off J Am Contact Dermat Soc 14(3):154–156. 10.2310/6620.2003.6169 10.2310/6620.2003.6169 14744407

[37] Malinauskiene L, Bruze M, Ryberg K, Zimerson E, Isaksson M (2010) Late patch test reaction to Disperse Orange 1 not related to active sensitization. Contact Dermatitis 63(5):298–299. 10.1111/j.1600-0536.2010.01810.x 20946466 10.1111/j.1600-0536.2010.01810.x

[38] Engfeldt M, Tillman C, Hindsén M, Bruze M (2012) Variability in patch test reactivity over time, falsely indicating patch test sensitization, in a patient tested with palladium salts. Contact Dermatitis 67(2):109–111. 10.1111/j.1600-0536.2012.02086.x 22775547 10.1111/j.1600-0536.2012.02086.x

[39] Bruze M, Ahlgren C, Isaksson M, Kroona L (2023) Late-appearing patch test reactions to carvone do not need to be signs of active sensitization. Contact Dermatitis 89(3):207–209. 10.1111/cod.14365 37315567 10.1111/cod.14365

[40] Bruze M, Hedman H, Björkner B, Möller H (1995) The development and course of test reactions to gold sodium thiosulfate. Contact Dermatitis 33(6):386–391. 10.1111/j.1600-0536.1995.tb02072.x 8706395 10.1111/j.1600-0536.1995.tb02072.x

[41] Frick-Engfeldt M, Isaksson M, Zimerson E, Bruze M (2007) How to optimize patch testing with diphenylmethane diisocyanate. Contact Dermatitis 57(3):138–151. 10.1111/j.1600-0536.2007.01197.x 17680861 10.1111/j.1600-0536.2007.01197.x

[42] Isaksson M, Lindberg M, Sundberg K, Hallander A, Bruze M (2005) The development and course of patch-test reactions to 2-hydroxyethyl methacrylate and ethyleneglycol dimethacrylate. Contact Dermatitis 53(5):292–297. 10.1111/j.0105-1873.2005.00705.x 16283908 10.1111/j.0105-1873.2005.00705.x

[43] Isaksson M, Bergendorff O, Hamnerius N et al (2023) Active sensitization to dimethylthiocarbamylbenzothiazol sulphide: An unexpectedly strong rubber contact allergen. Contact Dermatitis 88(6):472–479. 10.1111/cod.14311 36975130 10.1111/cod.14311

[44] Gefeller O, Pfahlberg AB, Uter W (2013) What can be learnt from nothing? A statistical perspective. Contact Dermatitis 69(6):350–354. 10.1111/cod.12112 23848408 10.1111/cod.12112

[45] Anon (2023) Commission Regulation (EU) 2023/1545 of 26 July 2023 amending Regulation (EC) No 1223/2009 of the European Parliament and of the Council as regards labelling of fragrance allergens in cosmetic products. J EU. (L 188/1).

[46] von Elm E, Altman DG, Egger M et al (2008) The strengthening the reporting of observational studies in epidemiology (STROBE) statement: guidelines for reporting observational studies. J Clin Epidemiol 61(4):344–349. 10.1016/j.jclinepi.2007.11.008 18313558 10.1016/j.jclinepi.2007.11.008

[47] Thyssen JP, Menné T, Schnuch A et al (2009) Acceptable risk of contact allergy in the general population assessed by CE-DUR–a method to detect and categorize contact allergy epidemics based on patient data. Regul Toxicol Pharmacol RTP 54(2):183–187. 10.1016/j.yrtph.2009.04.001 19383524 10.1016/j.yrtph.2009.04.001

[48] Schnuch A, Uter W, Geier J, Gefeller O, IVDK study group (2002) Epidemiology of contact allergy: an estimation of morbidity employing the clinical epidemiology and drug-utilization research (CE-DUR) approach. Contact Dermatitis. 47(1):32–39. 10.1034/j.1600-0536.2002.470107.x 12225411 10.1034/j.1600-0536.2002.470107.x

