# Supplementary material for: Extended fragrance ingredients surveillance study (EFISS)—protocol for a clinical surveillance study on contact allergy to 7 fragrance materials in widespread use but hitherto not systematically patch tested
Source: Arch Dermatol Res. 2025 May 23;317(1):778. doi: 10.1007/s00403-025-04286-9 (PMC12102106; doi:10.1007/s00403-025-04286-9)
Supplement: Supplementary file 1 — Supplementary file1 (PDF 967 KB) [file 403_2025_4286_MOESM1_ESM.pdf]

Case Report Form (CRF)

IDEA Extended Fragrance Ingredients Surveillance Study (EFISS):
Surveillance Study to Monitor the Frequency of Contact Allergy to a Defined Group of Fragrance Ingredients with a View to Providing Reliable Information on Trends

It should be noted that this is a paper representation of the eCRF and, for technical reasons, may not be a completely faithful reproduction of the eCRF. However, it is the primary record and data from it will be entered into the eCRF for analysis.

Study Centre: **IDEA Patient ID:**

Date of examination (YYYY):

Patient Details

Sex: ◯ **Male** ◯ **Female** ◯ **Unknown/Other**

◯ 18 - 20 ◯ 21-25 ◯ 26 -30 ◯ 31 – 35 ◯ 36-40 ◯ 41 – 45

◯ 46 – 50 ◯ 51 -55 ◯ 56 – 60 ◯ 61 – 65 ◯ 66 – 70 ◯ 71 -75

◯ 76 – 80 ◯ 81 – 85 ◯ 86 +

Occupation per ISCO.08. See list attached.

Code:

Exclusion/Exclusion criteria:

Subjects will be consecutive dermatitis patients. Subjects will be those attending established departments within the selected centres for routine diagnostic patch test investigations.

No specific exclusion criteria other those covered by the ESCD guidelines (*European Society of Contact Dermatitis guideline for diagnostic patch testing – recommendations on best practice, 2015*)

Study Centre: **IDEA Patient ID:**

Patient History

**Atopic eczema (Currently)**	**Yes** ◯	**No** ◯	**Unknown**◯
**Atopic eczema (Previously)**	**Yes** ◯	**No** ◯	**Unknown**◯

Present Dermatitis Leading to Current Testing

Onset of present dermatitis (MM/YY):

Localisation of this Dermatitis

Indicate locations:

**Hands**	◯
**Arms**	◯
**Trunk**	◯
**Neck**	◯
**Eyelids**	◯
**Scalp**	◯
**Face (Rest of)**	◯
**Legs**	◯
**Feet**	◯
**Ano/Genital area**	◯
**Axillae**	◯
**Other**	◯

Previous Fragrance-Related Dermatitis

Previously patch tested?

Unknown◯ **Yes** ◯ **No** ◯

If Yes, date (YYYY) previously tested: __________________________

Outcome of testing:

◯ **Yes, positive FM I**

◯ **Yes, positive FM II**

◯ **Yes, positive for other (Please specify):**

◯ **Yes, but tested negative to fragrances**

◯ **Unknown, e.g., patient cannot remember**

Study Centre: **IDEA Patient ID:**

Previous[1] dermatitis:

Yes ◯ **No** ◯

Onset of previous dermatitis (YYYY):

Resolution of previous dermatitis (YYYY):

[1] I.e., excluding “present” dermatitis as above

Study Centre: **IDEA Patient ID:**

Patch test preparation and performance

As standard practice, the test material should be stored at/below 4 degrees C prior to preparation, prepared less than 30 minutes before application and the test location should be on the back.

Study Centre: **IDEA Patient ID:**

Were Chemotechnique-supplied syringes containing both standard and Additional Materials used for the study? **Yes** ◯ **No** ◯

If not, was a detailed list of non-Chemotechnique materials used agreed in advance with the Study Managers?

Yes ◯ **No** ◯

Was this agreed list adhered to? **Yes** ◯ **No** ◯

If not detail any exceptions: ______________________________

Test location:

If test location other than back, please indicate location used:

____

Only for patients under immunosuppressive drug: please enter medication and dose:

Has the patient been advised that they should contact the centre should any reactions occur up to 28 days subsequent to the test application that they should contact the centre for re-evaluation?

Yes ◯ **No** ◯

Positive test outcome

Positive to at least one of the 7 additional fragrance allergen materials

Yes ◯ **No** ◯

In the event of a positive reaction for the additional materials only (Not required for standard materials) the EFISS Management Team should be contacted either by email or telephone/SMS.

Matthias Vey: mvey@ifrafragrance.org

+32 497 666206

Joseph Huggard: jh@huggardconsulting.com

+352 661 530613

Study Centre: **IDEA Patient ID:**

Patch Test Reading Sheet (A detailed sheet to be completed for all Additional Materials with positive reactions. Not required for standard materials)

	**Material**<br><br>**Additional Materials**	**First Reading**<br>**Day:**	**Second Reading**<br>**Day:**	**Reaction (Yes/No)**	**Detailed sheet completed Y/N**	**Comment**
31	Furaneol [4-Hydroxy-2,5-dimethyl-3(2H)-furanone)					
32	trans-2-Hexenal					
33	4,8-Dimethyl-4,9-decadienal					
34	Longifolene					
35	Benzaldehyde					
36	*Evernia prunastri* (Oak moss) with atranol and chloroatranol content at trace levels					
37	*Evernia furfuracea* (Treemoss) with atranol and chloroatranol content at trace levels					

	Material	First Reading<br>Day:	Second Reading<br>Day:	Reaction (Yes/No)	Relevance[2]<br>(See footnote)	Comment
	**Fragrance Mixes**					
1	Fragrance Mix I					
2	Sorbitan Sesquioleate					
3	Fragrance Mix II					
4	*Myroxylon pereirae* (Balsam of Peru) oil					
	**FM I Constituents**					
5	Amyl cinnamal					
6	Cinnamyl alcohol					
7	Cinnamal					
8	Eugenol					
9	Geraniol					
10	Hydroxycitronellal					
11	Isoeugenol					
12	*Evernia prunastri* (Oak moss extract)					
	**FM II Constituents**					
13	HICC (Hydroxyisohexyl 3-cyclohexene carboxaldehyde)					
14	Citral					
15	Citronellol					
16	Farnesol					
17	Coumarin					
18	Hexyl cinnamal					
	**Non-mix Fragrances**					
19	Amylcinnamyl alcohol					
20	Benzyl salicylate					
21	Anisyl alcohol					
22	Benzyl alcohol					
23	Benzyl cinnamate					
24	Butylphenyl methylpropional					
25	Linalool (non-ox.)					
26	Benzyl benzoate					
27	Limonene (non-ox.)					
28	Methyl 2-Octynoate					
29	Alpha-isomethyl ionone					
30	*Evernia furfuracea* (Treemoss extract)					

[2] For Relevance, enter CR (current relevance), PR (past relevance), UR (unknown relevance), XR (cross-reaction), NA (not allergic), and AS (active sensitisation),
