# Supplementary material for: Extended fragrance ingredients surveillance study (EFISS)—protocol for a clinical surveillance study on contact allergy to 7 fragrance materials in widespread use but hitherto not systematically patch tested
Source: Arch Dermatol Res. 2025 May 23;317(1):778. doi: 10.1007/s00403-025-04286-9 (PMC12102106; doi:10.1007/s00403-025-04286-9)
Supplement: Supplementary file 2 — Supplementary file2 (PDF 416 KB) [file 403_2025_4286_MOESM2_ESM.pdf]

Study Centre: **IDEA Patient ID:**

Detailed Sheet for <u>Additional Materials Only</u> (Enter material name for which a reaction was obtained – One material per sheet):

**Positive reaction to:**

Assessment as allergic versus irritant

◯ Allergic ◯ Irritant ◯ Uncertain

Relevance:

Select one of the following:

◯ current relevance ◯ past relevance ◯ unknown relevance

◯ cross reaction ◯ not allergic (irritant) ◯ active sensitisation

**Additional Comments/(Observations:**

Study Centre: **IDEA Patient ID:**

Morphology

Erythema

First Reading: Part of test area ◯ Whole of test area ◯

Second Reading: Part of test area ◯ Whole of test area ◯

Infiltration/Oedema

First Reading: Part of test area ◯ Whole of test area ◯

Second Reading: Part of test area ◯ Whole of test area ◯

Papules

First Reading: Part of test area ◯ Whole of test area ◯

Second Reading: Part of test area ◯ Whole of test area ◯

Vesicles/Bullae

First reading: Vesicles ◯ Bullae ◯

Second Reading: Vesicles ◯ Bullae ◯

Dry skin/Scaling

First reading: Dry skin ◯ Scaling ◯

Second Reading: Dry skin ◯ Scaling ◯

Pustules/Petechiae

First reading: Pustules ◯ Petechiae ◯

Second Reading: Pustules ◯ Petechiae ◯

Study Centre: **IDEA Patient ID:**

Erosion/Necrosis

First reading: Erosion ◯ Necrosis ◯

Second Reading: Erosion ◯ Necrosis ◯

Shiny skin/Silk paper structure

First reading: Shiny skin ◯ Silk paper ◯

Second Reading: Shiny skin ◯ Silk paper ◯

Other Morphology (Please specify):

Study Centre: **IDEA Patient ID:**

Product Identification Sheet (For each positive patch test reaction to one of the Additional Materials only, review with patient to determine if they believe a specific product to be associated with their reaction):

Patient associates his/her dermatitis with a specific product **Yes** ◯ **No** ◯

Identified Fragrance Ingredients (List):

Details of product identified (Complete the following table to the fullest extent possible, based on patient information)

Product type	Personal Use	Occupational Use	Product Details (Brand, product name, additional identifiers e.g., batch number, anti-bacterial).
**Creams/Lotions**			
-body			
-face			
-eyes			
-hands			
-feet			
**Sunscreens**			
**Self-tanning**			
**Deodorant**			
-spray			
-roll-on/stick			
**Make-up**			
-rinse off face masks			
-eye make-up			
-tinted bases			
-make-up remover			
**Wet wipes**			
**Hairstyling**			
-gels/mousse			
-sprays			
**Mouths washes**			
**Rinse-off cosmetics**			
-liquid soap			
-bath/shower gel			
-shampoo			
-conditioner			
**Shaving products**			
**Cleaning agents**			
**Household cleaning spray**			
**Dishwashing liquid**			
**Aromatherapy**			
**Others (Please list):**			
